# The Principles of Diagnosis. Part the Second. The Diagnosis of the More General Diseases of Adults

**Published:** 1818-04

**Authors:** 


*The Principles of Diagnosis. Part the Second. The Diagnosis of the more general Diseases of Adults.* By Marshal Hall, M. D. *One vol. octavo. pp.* 339. *with numerous tables.*

This is one of the most extraordinary works we have ever perused. After nearly thirty years of experience we are conscious, that had we noted and arranged every case and every symptom that presented themselves in all that time, we could not have furnished one fourth the quantum of matter presented in this volume. On the other hand, if these diagnostics were compiled from books, the labour must have been prodigious. Whether Dr. Hall shall have contributed to the practical advancement of nosology by his arrangements, and very minute distinctions, we shall not venture to predict; but certainly he has imposed on himself a most Herculean task in the present undertaking; and if he goes through it with the same minuteness as is here displayed, he may undertake any thing in future.

"The present volume, which constitutes the *second* of the work, is the *first* part of the diagnosis of diseases, and is published in the first place, to afford the *order* for treating the subjects of the volume by which it is to be *preceded.*"

There is undoubtedly some want of *order* in this passage, or else our intellects are unusually dull; but this is not of much consequence. Dr. Hall appears, from a passage in the Introduction, to have selected the subject of *diagnosis* for a life of medical study and observation, and to introduce a suite of *cases* illustrative of the different characters of disease, and which may obviate the objection to systematic works in general. On this point we fully agree with our author, and believe, that elucidations of this kind are often more useful and impressive than the most laboured descriptions. In this work, Dr. Hall informs us, that

"— diseases of general similitude, and therefore of obscure diagnosis, are classed together, in order that the *difficulties* in their discrimination may be represented, and that the *means* of their discrimination may be more effectually compared and contrasted." p. 8.

This work, unlike many, is totally incapable of being regularly analysed; the matter being in a state of great concentration already, and the subjects extremely diversified. We must therefore select a single section, and by laying a very full portrait of it before the reader, do justice to the author himself, and give a sufficient specimen of the executionof the work.

*Section the Third.* ACUTE DISORDERS OF THE DIGESTIVE ORGANS.

1. *In early youth.* Acute disorder of the digestive apparatus in this period, is characterized by transient flushings of the cheeks, alternating with paleness, sallowness, and indisposition. Eyes dull; eyelids heavy; pupils frequently large. The little patient appears languid, dull, listless, dejected; often lying long in one position without moving, speaking, or taking notice. He frequently picks the nose; skin is often hot and dry; and at length becomes shrivelled, rough, and exfoliates; occasionally cool and partial perspirations. Emaciation takes place; more observable about the body and limbs than in the countenance. Tongue is loaded; breath fœtid; headaches; sometimes stupor; sometimes a fixed stare or rolling of the eyes; sometimes dimness or loss of sight. Sleep is disturbed with starting, screaming, moaning, or grinding of the teeth. Pulse frequent; appetite various; generally impaired, sometimes craving or voracious; occasional vomiting. Bowels at first slow and costive; afterwards constipation alternates with pain and diarrhœa. Flatulence; tumid abdomen; with pain fixed or wandering; stools fœtid, dark coloured, clay-like, slimy, or mixed, with sometimes worms. Urine generally turbid on standing, with copious sediment.

This affection often commences insidiously, and continues from one month to an unlimited period, frequently assuming the form of chronic disorder of the digestive organs about to be described. It has been denominated worm fever; infantile hectic, or slow fever; infantile remittent fever; and marasmus. Vide, Butter, Pemberton, Hamilton, and Dr. Armstrong.

The disease is complicated with, 1st. Affections of the head. 2d. Spasmodic or convulsive affections. 3d. Hydrencephalus. 4. Pain and tenderness of the abdomen. 5. Inflammation of the abdomen.

2. *Chronic Disorder of the Digestive Organs.* This is characterized, in early youth, by paleness of the countenance, sometimes resembling the colour of light tea paper; sometimes a leaden hue; slight flush on the cheeks occasionally; eyelids surrounded with a dark brown ring; upper lip tumid; both lips exsanguious; pupils often large; tongue clean; breath not tainted; debility; irritability; listlessness; chilliness; great emaciation of the arms, hands, and body; skin dry and shrivelled; irregular cold perspirations; swellings of the ankles in the ulterior periods; head less affected than in the acute form of the disease; pulse frequent; frequently pain in the abdomen, with diarrhœa; little appetite, with peculiar cravings.

This state sometimes succeeds the acute, sometimes comes on in the most slow and insidious manner. It continues for many months, sometimes years, ultimately proving fatal. It often terminates in organic disease of some of the important viscera of the abdomen, thorax, or head. It ought to be distinguished from disease of the mesenteric glands, and enlargement of the liver.

*Causes.* Confinement, and sedentary life; crowded streets, low and unwholesome situations; impure air; filth; poor indigestible diet.

### In Youth and Adult Age.

"Acute disorder of the digestive apparatus in youth or adult age, is generally attended with a pallid and sallow state of the countenance, which is, however, very variable, and easily flushed by warmth, coughing, the presence of a stranger, or any mental agitation. A ring of darkness soon forms round the eyes. Sometimes the face seems covered with a viscid and oily perspiration, a circumstance which often accompanies and denotes a loaded state of the tongue. The tongue is loaded in every case; sometimes it is black at the posterior part, and white nearer the point; it is in general moist; and the papillæ are sometimes much elongated. The breath is much tainted. There are languor, debility, and nervousness; fatigue and pain of the side on attempting to use exercise. There is early emaciation, much more manifest in the limbs and about the trunk, than in the face, which appears sickly, but is not soon observed to become thin. The skin becomes shrivelled, and exfoliates slightly; it is occasionally hot, generally dry, but there is a degree of perspiration sometimes. There is generally head-ache; loss of the power of attention and of memory; irritability of temper; lowness of spirits. The pulse is frequent, from one hundred to one hundred and twenty, and easily accelerated. The appetite is impaired; there is fastidium or loathing; sometimes the sight of food cannot be endured; sometimes a particular article of diet is relished, and desired continually, as cheese, pickles, water cresses, &c. Improper food taken, excites vomiting, or pain and a sense of load in the stomach, or pain of the bowels and diarrhœa, and aggravates all the other symptoms. The bowels are constipated; the alvine evacuation dark, fœtid, flatulent, or otherwise unnatural. The urine deposits a very copious sediment.

"There are also some other circumstances which attend and denote this state of disorder of the digestive organs. The affection is multiform, and exceedingly variable; the patient is better and worse, sometimes apparently almost well, and soon again exceedingly indisposed. There are often vertigo and affection of the head; dyspnœa, palpitation, or cough; pain of the abdomen, diarrhœa; symptoms which occur in fits, and induce hurry and agitation. The cure is thus interrupted, and is at length effected slowly, and often imperceptibly. p. 104.

The reader will perceive from this quotation, that Dr. Hall is led into perpetual tautology by narrating the *minor* symptoms which are common to a variety of diseases, instead of dwelling on the *distinctive* features of each. And this minute detail completely frustrates the object of diagnosis, as far as relates to the *memory*; and even on reference to the whole symptomatology, it will be no easy task for the clinical student to separate or distinguish the diagnostic symptoms of a disease from those which are common to it and many others. Indeed, we think Dr. Hall has chosen a very bad title for his work; for instead of "Principles of *Diagnosis*," it should have been, "Minute *Symptomatology* of Diseases."

A long and minute description of *Chlorosis* follows, which we do not deem it necessary to analyse. It appears from Dr. Hall, that

"Chlorosis is endemic at Nottingham, and other manufacturing towns, where a great part of the population is engaged from morning till night in the sedentary occupations of seaming, lace-running, of the tambour, &c." p. 109.

*Disorder of the Digestive Organs in advanced Age.*

This is less distinctly characterized than in youth. The tongue is loaded; the breath tainted; bowels constipated, or irregular; head-ache; vertigo; disturbed sleep; nervousness; dyspnœa; irregular action of the heart, &c. in different instances.

*Complications.* 1. With symptoms of affection of the head. 2. Threatening of apoplexy. 3. Derangement of the mind. 4. Pain of the chest, irregular action of the heart, &c. 5. Angina pectoris. 6. Constipation followed by diarrhœa, faintness, irregular pulse, &c.

From the sections on hysteria and hypochondriasis, we cannot glean any thing novel or interesting, and the specimens we have already offered will be sufficient to give the reader an idea of the work.

At page 306—7, we meet with two cases of tic douloureux successfully treated by arsenic. One of these we shall transcribe:—

"May 19th, 1817. Miss R. aged 20, on the 10th instant, about 11, A. M. she became affected, without assignable cause, with acute pain under the right eyebrow, which continued fifteen minutes, and then ceased. It returned, for a short time, about 7, P. M. Since this day, the pain has returned daily about 10, or half past 10, A. M. and about 7, P. M.; it has become daily more and more severe and excruciating, and it now induces at each paroxysm, suffusion of the eyes and eyelids, a profuse flow of tears, down the cheeks, and into the nostrils; with a degree of intolerance of light; and, from the severity of the pain, a tremulous agitation of the whole body; the paroxysm continues, in the morning, until about 2, P. M. and in the evening, about an hour and a half; it is apt to be succeeded by a tendency to sleep. On the 12th, a dose of opening medicine was prescribed. On the 13th, the patient was recommended a gentle aperitive each night, and six drops of Fowler's solution of arsenic at 9, A. M. and at 9, P. M. On the 15th, the pain was *most* excruciating; the eyes suffused, and tender on exposure to the light, the eye-lids red and swollen; the tears flowed copiously, inducing a constant use of the handkerchief; the pain extended from the inner part of the eye-brow, high up the forehead; and the body was in a state of constant agitation. But the pulse was not accelerated. From this date the returns of the paroxysms were regular, but their duration and severity diminished daily, and they induced daily less redness, suffusion, and tears. During this affection, the tongue was slightly white, with a slight appearance of red points at its extremity. The catamenia were perfectly regular. The bowels kept open. This patient has a slightly decayed tooth, the third of the upper jaw of the contrary side to that of the pain. It was not extracted. May 16th. Since the last report the pain scarcely returned in the evening. On the 24th there was no pain. On the 25th, a slight degree of pain in the morning. On and since the 26th no pain. The medicine has been continued." P. 307.

Of the utility of the "*tables of diagnosis*" which Dr. Hall has constructed, as well as of that of the whole work, we dare scarcely venture an opinion. We sincerely wish it may answer the expectations of the author. We are quite sure, however, that were he to adopt the following suggestion in a future edition, the value of the work would be infinitely enhanced. We would term the work—"The symptomatology of diseases, with their diagnoses and causes." At the foot of each section we would arrange the *diagnostic* symptoms in contrast with those which are common to various diseases; and lastly, add the etiology, and perhaps a short *Methodus Medendi*, in the manner of Darwin. As the arrangement now stands, there appears to us to be no *diagnosis*, strictly speaking, at all. We will refer to rheumatism and gout—two diseases that are often confounded together, and yet require considerable modification of treatment. At page 314, the symptomatology of acute rheumatism is dispatched in twenty-five lines, and not a single word is said respecting its diagnosis. At page 316, acute gout occupies thirty lines, and no diagnostic marks are even alluded to. Now we would ask Dr. Hall, where the "principles of diagnosis" are to be sought for? If in the common symptomatology, we have much fuller histories of gout and rheumatism than he has presented us; and surely the practitioner, who finds himself in doubt respecting two diseases that simulate each other so much as these do, and refers to a work on diagnosis to solve the difficulty, must feel very much disappointed in the book before us.

We can assure Dr. Hall, that these objections are urged solely with the view of serving him; as experience has rendered us, in some degree at least, acquainted with the best, as well as the most profitable modes of laying medical information before the public. Dr. Hall appears to be a zealous and indefatigable observer; and if his exertions are turned into a proper channel at the beginning, the tide may lead him to reputation and fortune. Our best wishes attend him.

